# The COMET initiative database: progress and activities update (2014)

**DOI:** 10.1186/s13063-015-1038-x

**Published:** 2015-11-11

**Authors:** Elizabeth Gargon, Paula R. Williamson, Doug G. Altman, Jane M. Blazeby, Mike Clarke

**Affiliations:** Department of Biostatistics, University of Liverpool, Block F Waterhouse Building, 1-5 Brownlow Street, Liverpool, L69 3GL UK; Centre for Statistics in Medicine, Botnar Research Centre, University of Oxford, Windmill Road, Oxford, OX3 7LD UK; School of Social and Community Medicine, University of Bristol, Canynge Hall, 39 Whatley Road, Bristol, BS8 2PS UK; Queens University Belfast, Institute of Clinical Sciences, Block B, Royal Hospitals, Grosvenor Road, Belfast, BT12 6BA UK

**Keywords:** Core outcome set, database, resources

## Abstract

The COMET Initiative database is a repository of studies relevant to the development of core outcome sets (COS). Use of the website continues to increase, with more than 16,500 visits in 2014 (36 % increase over 2013), 12,257 unique visitors (47 % increase), 9780 new visitors (43 % increase) and a rise in the proportion of visits from outside the UK (8565 visits; 51 % of all visits). By December 2014, a total of 6588 searches had been completed, with 2383 in 2014 alone (11 % increase). The growing awareness of the need for COS is reflected in the website and database usage figures.

## Findings

### Background

The Core Outcome Measures in Effectiveness Trials (COMET) website and database were launched in August 2011, and the progress and activities up to 31 December 2013 were reported in *Trials* last year [1]. This letter outlines subsequent progress in 2014 (Source of data usage: Google Analytics). It provides data on the value and use of the COMET materials and on the interest in core outcome sets (COS) above and beyond what might be gleaned through, for example, data on the citation of key articles. COS represent the minimum outcomes that should be measured and reported in all clinical trials of a specific condition and may also be suitable for use in other types of research and clinical audit [2].

### Activity and content

**Fig. 1:**
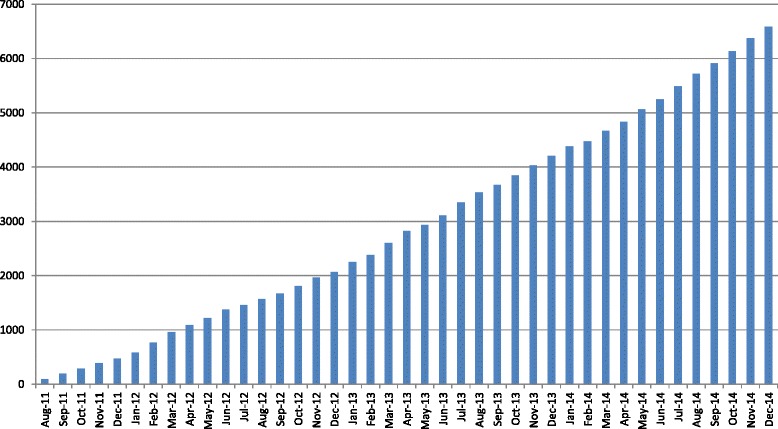
Cumulative number of completed searches in the COMET database (2011-2014)

On 31 December 2014, 567 studies relevant to the development of COS were included in the COMET database, up from 306 at the end of 2013. These included a total of 80 planned and ongoing studies, and the database had been boosted considerably by the addition of studies identified through a systematic review of core outcome sets that identified 198 published COS [3]. Usage statistics show that the number of visits increased from 12,332 during 2013 to 16,768 in 2014: a 36 % increase. The number of unique visitors increased by 47 % from 8369 in 2013 to 12,257 in 2014, and the number of new visitors, by 43 % from 6844 in 2013 to 9780 in 2014. Full details are provided in Table 1. There was a 38 % increase in page views from 2013 to 2014 (53,226 to 73,617 page views). By December 2014, a total of 6588 searches of the database had been run (Fig. 1), with 2383 in 2014 alone. The sustained growth in use suggests that the COMET website and database are continuing to gain interest and prominence and that they are an effective resource for people interested in core outcome set development.

**Table 1:** Usage statistics 2013-2014

	Number of visits				Number of unique visitors				Number of new visitors			
	2011	2012	2013	2014	2011	2012	2013	2014	2011	2012	2013	2014
January	-	670	1069	1282	-	450	657	985	-	385	542	842
February	-	762	1017	1052	-	463	648	849	-	378	525	736
March	-	649	1238	1221	-	429	761	942	-	358	617	406
April	-	683	1050	1244	-	466	678	961	-	395	564	831
May	-	659	1088	1113	-	407	721	774	-	330	504	622
June	-	435	1403	1043	-	305	887	714	-	260	703	569
July	-	472	945	1203	-	314	650	783	-	241	526	614
August	804	457	833	1673	503	324	576	1160	494	273	480	969
September	448	483	901	1604	314	347	623	1146	286	288	524	942
October	460	669	984	1832	295	516	802	1365	258	441	689	1135
November	686	1117	966	2260	484	854	727	1621	437	757	619	1338
December	580	926	838	1241	409	596	639	957	363	505	551	776
	2978	7982	12332	16768	2005	5471	8369	12257	1838	4611	6844	9780

As in previous years, most visits to the website were direct or via a search engine. Thirteen percent of all visits in 2014 were referrals, including Twitter (15 %), The Italian Cochrane Centre (6 %), MRC Network of Hubs for Trials Methodology Research (6 %), The University of Liverpool (5 %), BJOG: An International Journal of Obstetrics and Gynaecology (5 %), and Nature (4 %). The COMET IV meeting was jointly hosted by the Italian Cochrane Centre in Rome in November 2014, which reflects the large group of referrals from the Italian Cochrane Centre website; demonstrating how effective collaborative efforts can promote COMET. The Nature referrals can be explained by an editorial in Nature Medicine in August 2014 [4], demonstrating the impact of this type of high profile exposure.

Analyses of the COMET website data show that 57 % of visitors went beyond the page on which they landed. As in previous years, the most common first interaction was to complete a search in the COMET database. Other first interactions included moving to the page providing an overview of the COMET Initiative, accessing the database but without completing a search, and visiting the pages containing details of the COMET IV meeting or the COMET resources page. The Core Resource Pack is the second most highly accessed resource on the website (after the database), with 1064 page views in 2014, compared to 780 in 2013.

The content of the website continues to be updated regularly. In 2014, we extended the patient and public involvement resources beyond the Plain Language Summary that was available. There is now a Delphi Process Plain Language Summary, along with a Public Involvement Strategy outlining the COMET public involvement objectives and plans. In 2014, the plain language summary page was visited 301 times, and the Public Involvement page was visited 138 times since it was launched in August to December 2014.

The number of countries represented by visitors increased from a total of 113 in 2013 to 123 in 2014. A list of the 123 countries represented by visitors to the website in 2014 is shown in Appendix 1. This increase in the international use of the website and database is also reflected in the proportion of visits. In 2013, 59 % of the visits were from the United Kingdom (7256 of 12,332 visits). In 2014, the percentage of visits from the UK decreased to 49 % (8203 of 16768 visits), whereas visits from the United States and Canada rose to 16 % (from 12 % in 2013) and visits from the rest of the world increased to 35 % (29 % in 2013). This increase in visits from countries outside the UK reinforces COMET as an international initiative and demonstrates an increased global awareness and interest in core outcome sets and the COMET Initiative. Table 2 shows the ten countries with the most visits to the COMET website from 2012 to 2014. The presence of Japan in 2013 and India in 2014 reflects where COMET activities were undertaken, including the COMET workshop in Kyoto (2013) and the presence of the Cochrane Colloquium in Hyderabad (2014) [4]. This highlights the importance of international dissemination, but it is worth noting that all content and materials are provided in the English language only at the moment, and there are no immediate plans for translation.

**Table 2:** Countries represented by the most visits to the COMET website in 2012, 2013, and 2014

2012		2013		2014	
United Kingdom	5,577	United Kingdom	7,526	United Kingdom	8,203
United States	431	United States	1,022	United States	2,038
Canada	326	Canada	501	Italy	1,115
Australia	201	Australia	321	Canada	624
Germany	186	Italy	315	Germany	581
Netherlands	166	Netherlands	308	Netherlands	510
Italy	161	Germany	285	Australia	494
France	125	Japan	228	France	374
Ireland	113	France	227	India	306
Norway	62	Ireland	159	Ireland	239

As noted above, 6588 searches have been completed in the database since its launch in August 2011 to December 2014, with 2383 in 2014. The search allows the user to take a structured approach to finding COS, and the most frequently used search criteria in 2014 were consistent with previous years. Disease category (74 %) was the most frequently used, followed by disease name (47 %), study type (30 %), type of intervention (26 %), methods used (25 %), and stakeholders involved (24 %). The most commonly searched terms were ‘cancer’ (n = 129), ‘mental health’ (n = 116), ‘pregnancy and childbirth’ (n = 86), and ‘neurology’ (n = 82).

### Plans for the future

An update of our systematic review of core outcome sets [2] is planned for early 2015. This will help to keep the database up to date and ensure that it is an effective resource for users. As before, we continue to identify and include studies in an ad hoc way to keep the database current. A pop-up survey is planned for 2015 to gather information from users in order to evaluate how and why people are using the database. This will allow us, for example, to consider ways to improve the search functions available. Other activities for 2015 include the first COMET meeting (COMET V) to be held outside of Europe, in Calgary, Canada, in May. Finally, we plan to expand the patient and public involvement resources available on the website, and this will be a priority for the newly formed COMET Patient and Public Involvement (PPI) working group.

The COMET website and database usage figures will continue to be monitored and assessed annually.

## Appendix 1

**Table 3:** Countries represented by the site visitors in 2014 (n = 123)

Algeria	Iceland	Philippines
Argentina	India	Poland
Australia	Indonesia	Portugal
Austria	Iran	Puerto Rico
Bahamas*	Iraq	Qatar
Bahrain	Ireland	Romania
Bangladesh	Israel	Russia
Belarus	Italy	Saudi Arabia
Belgium	Jamaica	Serbia
Bhutan*	Japan	Singapore
Bolivia*	Jersey	Slovakia
Bosnia & Herzegovina	Jordan	Slovenia
Botswana*	Kazakhstan	South Africa
Brazil	Kenya	South Korea
Bulgaria	Kuwait	Spain
Cameroon	Latvia	Sri Lanka
Canada	Lebanon	Sudan
Cayman Islands	Lithuania	Swaziland*
Chile	Luxembourg	Sweden
China	Macau	Switzerland
Colombia	Macedonia (FYROM)*	Syria*
Costa Rica	Madagascar*	Taiwan
Croatia	Malawi	Tanzania
Cyprus	Malaysia	Thailand
Czech Republic	Maldives	Trinidad & Tobago
Denmark	Malta	Tunisia*
Dominican Republic	Mauritius*	Turkey
Ecuador	Mexico	Uganda
Egypt	Montenegro*	Ukraine
Estonia	Morocco	United Arab Emirates
Ethiopia	Myanmar (Burma)	United Kingdom
Finland	Namibia*	United States
France	Nepal	Venezuela
Gambia*	Netherlands	Vietnam
Georgia	New Zealand	Yemen
Germany	Nigeria	Zambia
Ghana*	Northern Mariana Islands*	Zimbabwe
Greece	Norway	
Guadeloupe*	Oman	
Haiti*	Pakistan	
Honduras*	Palestine*	
Hong Kong	Panama*	
Hungary	Peru	

*New to 2014

## Abbreviations

COMET: Core Outcome Measures in Effectiveness Trials
COS: core outcome set(s)
